# Effects of age and gender on the pharmacokinetics of the soluble guanylate cyclase stimulator riociguat

**DOI:** 10.1186/2050-6511-14-S1-P23

**Published:** 2013-08-29

**Authors:** Reiner Frey, John Lettieri, Andrea Nadel, Corina Becker, Wolfgang Mück

**Affiliations:** 1 Clinical Pharmacology, Bayer Pharma AG, Pharma Research Centre, Wuppertal, Germany; 2 Clinical Pharmacology, Bayer HealthCare Pharmaceuticals, Montville, NJ, USA; 3 Clinical Statistics, Bayer HealthCare Pharmaceuticals, Montville, NJ, USA

## Background

Pulmonary hypertension is a disabling disease associated with high mortality [1,2]. Riociguat (under review for the treatment of pulmonary hypertension) stimulates soluble guanylate cyclase, which plays an important role in the regulation of cardiovascular tone and remodelling [3-9]. We investigated the potential effects of age and gender on the pharmacokinetics of riociguat and its primary metabolite M1 (BAY 60-4552). Safety and tolerability of riociguat were also assessed.

## Methods

This placebo-controlled, double-blind, single-centre study followed good clinical practice guidelines. Healthy volunteers were randomized into four groups according to age (young, 18–45 years; elderly, 64.5–80 years) and gender: young male (YM), elderly male (EM), young female (YF), elderly female (EF). All participants received a single oral tablet of riociguat 2.5 mg or placebo. Dense sampling was performed for pharmacokinetics.

## Results

Forty-seven participants provided data for pharmacokinetic and safety analyses. Nine participants in each group received riociguat; three participants in each of the YM, EM and EF groups and two participants in the YF group received placebo.

**Figure 1:**
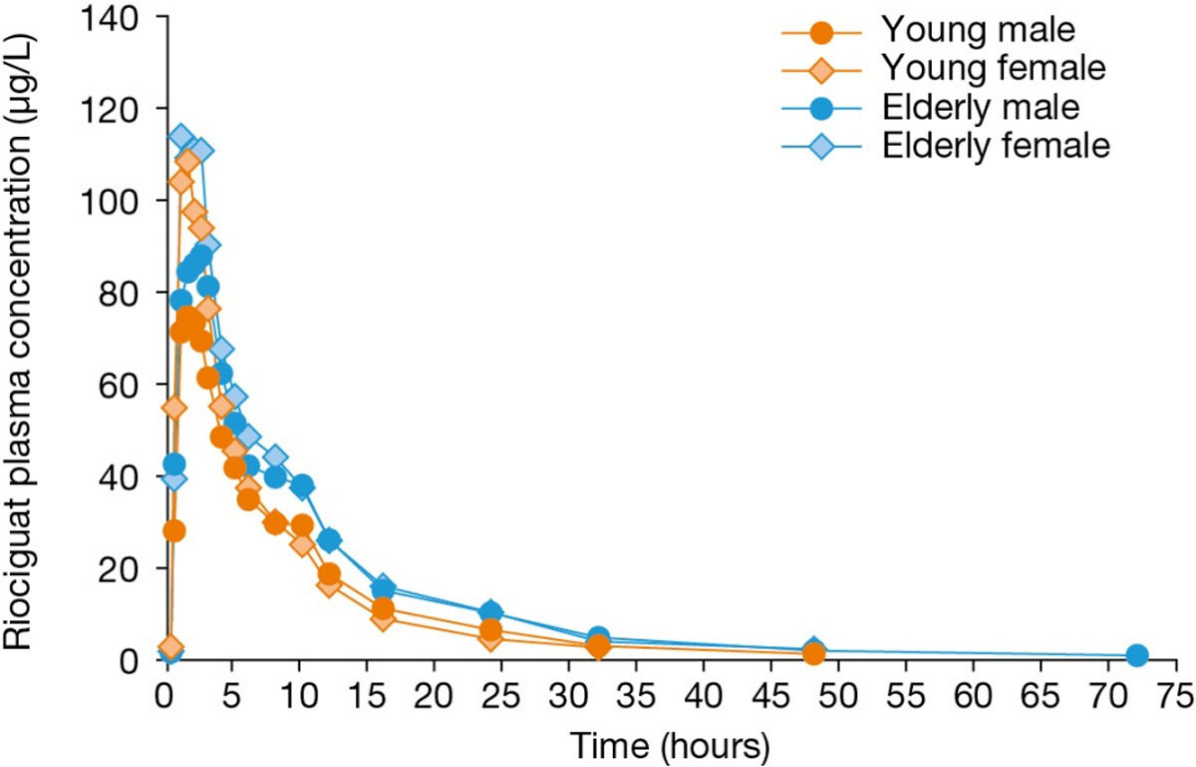
Mean riociguat plasma concentrations in groups of differing age and gender following administration of a single oral dose of riociguat 2.5 mg.

Age: the mean maximum concentration of riociguat in plasma (C_max_) did not vary markedly between age groups (Figure 1). However, mean renal clearance was decreased in the elderly, and exposure (area under the plasma concentration–time curve [AUC]) to riociguat was approximately 40% higher in the elderly than in the young (p > 0.05) (Tables 1 &2). When normalized for body weight, the riociguat exposure (AUC_norm_) ratio was reduced; AUC_norm_ was approximately 30% higher in the elderly than in the young (Tables 1 &2).

**Table 1:** Selected pharmacokinetic parameters of riociguat and metabolite M1 (BAY 60-4552) by age and gender following a single oral dose of riociguat 2.5 mg

Parameter	Young female (n = 9)	Elderly female (n = 9)	Young male (n = 9)	Elderly male (n = 9)
Riociguat				
AUC, μg·h/L	803 (55)	1145 (36)	750 (58)	1036 (48)
C_max_, μg/L	113 (22)	125 (35)	80.4 (25)	96.7 (32)
AUC_norm_, g·h/L	24.8 (57)	32.6 (45)	26.5 (55)	32.8 (50)
C_max,norm_, g/L	3.48 (20)	3.56 (36)	2.84 (23)	3.06 (22)
t_½_, h	8.97 (51)	11.8 (31)	8.10 (39)	12.2 (62)
CL_R_, L/h^a^	0.320 (28)	0.223 (33)	0.389 (37)	0.287 (28)

M1				
AUC, μg·h/L	640 (26)	651 (38)	476 (20)	687 (44)
C_max_, μg/L	28.1 (53)	22.4 (52)	14.9 (44)	18.8 (71)
AUC_norm_, g·h/L	20.5 (26)	19.2 (30)	17.4 (28)	22.5 (35)
C_max,norm_, g/L	0.899 (51)	0.661 (42)	0.544 (51)	0.615 (65)
t_½_, h	16.0 (34)	16.7 (21)	14.9 (28)	21.3 (33)
CL_R_, L/h^a^	0.824 (32)	0.487 (36)	0.718 (39)	0.573 (44)

^a^Arithmetic mean (percentage coefficient of variation). All other parameters are expressed as geometric means (percentage coefficient of variation).

AUC, area under the plasma concentration–time curve from time 0 to infinity; AUC_norm_, AUC divided by dose per kilogram of body weight; C_max_, maximum concentration in plasma; C_max,norm_, C_max_ divided by dose per kilogram of body weight; t_½_, terminal elimination half-life; CL_R_, renal clearance.

**Table 2:** Comparison of selected riociguat and M1 pharmacokinetic parameters between different participant groups (ratios and 90% confidence limits)

Parameter	Elderly vs young (n = 18 per group)	Female vs male (n = 18 per group)
Riociguat		
AUC	1.40 (1.06–1.86)	1.09 (0.82–1.44)
C_max_	1.16 (0.98–1.36)	1.35 (1.14–1.59)*
AUC_norm_	1.28 (0.95–1.71)	0.97 (0.72–1.30)
C_max,norm_	1.05 (0.91–1.22)	1.20 (1.03–1.38)*
t_½_	1.41 (1.08–1.84)*	1.04 (0.79–1.35)

M1		
AUC	1.21 (1.00–1.46)	1.13 (0.94–1.36)
C_max_	1.00 (0.73–1.38)	1.50 (1.09–2.05)*
		
AUC_norm_	1.10 (0.93–1.30)	1.00 (0.85–1.19)
C_max,norm_	0.91 (0.68–1.23)	1.33 (0.99–1.79)
t_½_	1.22 (1.04–1.44)*	0.92 (0.78–1.08)

*p < 0.05 for ratio = 1.

AUC, area under the plasma concentration–time curve from time 0 to infinity; AUC_norm_, AUC divided by dose per kilogram of body weight; C_max_, maximum concentration in plasma; C_max,norm_, C_max_ divided by dose per kilogram of body weight; t_½_, terminal elimination half-life.

Gender: although riociguat mean C_max_ normalized for body weight (C_max,norm_) was significantly greater in women than in men, no difference in exposure was observed between genders (Tables 1 &2).

Across age groups, pharmacokinetics of M1 followed similar trends to those of riociguat, although differences were less pronounced (Tables 1 &2). Compared with the combined placebo subgroups, the combined riociguat subgroups demonstrated an expected reduction in mean blood pressure and corresponding elevation of mean heart rate, waning approximately 16 hours post-dose. Three participants in the riociguat subgroups reported drug-related adverse events, one of which (hypotension) was classified as severe. All adverse events had resolved by completion of the study.

## Conclusion

Age and gender had modest effects on riociguat and M1 pharmacokinetics, and the safety profile of riociguat was similar across all groups. Thus, no dose adjustment for age or gender is merited.

## References

[B1] D'Alonzo GE Barst RJ Ayres SM Bergofsky EH Brundage BH Detre KM Fishman AP Goldring RM Groves BM Kernis JT Survival in patients with primary pulmonary hypertension. Results from a national prospective registry Ann Intern Med 1991 115 343 349 10.7326/0003-4819-115-5-343 1863023

[B2] Hurdman J Condliffe R Elliot CA Davies C Hill C Wild JM Capener D Sephton P Hamilton N Armstrong IJ Billings C Lawrie A Sabroe I Akil M O'Toole L Kiely DG Aspire Registry: assessing the spectrum of pulmonary hypertension identified at a referral centre Eur Respir J 2012 39 945 955 10.1183/09031936.00078411 21885399

[B3] Stasch JP Pacher P Evgenov OV Soluble guanylate cyclase as an emerging therapeutic target in cardiopulmonary disease Circulation 2011 123 2263 2273 10.1161/CIRCULATIONAHA.110.981738 21606405 PMC3103045

[B4] Schermuly R Stasch JP Pullamsetti SS Middendorff R Mueller D Schlüter KD Dingendorf A Hackemack S Kolosionek E Kaulen C Dumitrascu R Weissmann N Mittendorf J Klepetko W Seeger W Ghofrani HA Grimminger F Expression and function of soluble guanylate cyclase in pulmonary arterial hypertension Eur Respir J 2008 32 881 891 10.1183/09031936.00114407 18550612

[B5] Frey R Mück W Unger S Artmeier-Brandt U Weimann G Wensing G Single-dose pharmacokinetics, tolerability and safety of the soluble guanylate cyclase stimulator BAY 63-2521; an ascending-dose study in healthy male volunteers J Clin Pharmacol 2008 48 926 934 10.1177/0091270008319793 18519919

[B6] Ghofrani HA Hoeper MM Halank M Meyer FJ Staehler G Behr J Ewert R Weimann G Grimminger F Riociguat for chronic thromboembolic pulmonary hypertension and pulmonary arterial hypertension: a phase II study Eur Respir J 2010 36 792 799 10.1183/09031936.00182909 20530034

[B7] Ghofrani H Grimminger F Hoeper M Kim N Mayer E Neuser D Pena J Simonneau G Wilkins M Riociguat for the treatment of inoperable chronic thromboembolic pulmonary hypertension: a randomized, double-blind, placebo-controlled study (CHEST-1) Chest 2012 142 1023A 10.1378/chest.1462924

[B8] Ghofrani H Galie N Grimminger F Humbert M Keogh A Langleben D Kilama MO Neuser D Rubin L Riociguat for the treatment of pulmonary arterial hypertension: a randomized, double-blind, placebo-controlled study (PATENT-1) Chest 2012 142 1027A 10.1378/chest.146279923032451

[B9] Hoeper MM Halank M Wilkens H Gunther A Weimann G Gebert I Leuchte H Behr J Riociguat for interstitial lung disease and pulmonary hypertension: a pilot trial Eur Respir J 2013 41 853 860 10.1183/09031936.00213911 22936711

